# Improving Patient Handoffs and Transitions in Care Among Residents: A Chief Resident-Led Initiative

**DOI:** 10.7759/cureus.73282

**Published:** 2024-11-08

**Authors:** Mariel Marquez, Athena Gonzalez, Youmna Moufarrej, Vini Vijayan

**Affiliations:** 1 Pediatrics, Valley Children's Healthcare, Madera, USA; 2 Medical Education, Valley Children's Healthcare, Madera, USA; 3 Pediatrics, Children's Hospital Los Angeles, Los Angeles, USA

**Keywords:** handoff, i-pass, medical education, patient safety, residents, transfer of care

## Abstract

Introduction: Effective handoff between pediatric residents is crucial to ensure continuity of care and patient safety. Omissions in information and communication breakdowns can be associated with uncertainty in clinical decision-making and adverse patient events. In our role as chief residents, we were notified of an increase in patient safety alerts due to communication failures and gaps during handoff. We aimed to identify areas for improvement and implement strategies to improve competence in handoff among pediatric residents. We also explored pediatric residents' confidence levels regarding handoff procedures and the effectiveness of our interventions in the transfer of care.

Methods: Two chief residents conducted direct handoff observations of residents during the transfer of care of inpatients over six months. Residents were scored using a handoff checklist, and formative feedback was provided to each resident after the observation session. Deficits and barriers to properly executed handoff were noted and used to develop a series of handoff workshops. Pre- and post-workshop confidence in handoff skills was calculated from an average of each five-point Likert scale item (1=not at all confident, 5=very confident).

Results: Forty pediatric residents were assessed performing inpatient handoff. We observed 38 handoff sessions. All of these involved face-to-face interactions with verbal and written communication in the I-PASS (illness severity, patient summary, action list, situation awareness and contingency planning, and synthesis by the receiver) format, allowing the receiver of the information to clarify issues and ask questions. Protocol failures were identified in 50% of the handoffs observed. This included disruptions during handoff (5%), incorrect relay of patient information (26%), prioritizing sick patients (26%), omission of care tasks (10%), and provision of contingency planning (31%). Forty residents participated in the handoff workshops.

Regarding confidence in handoff before and after the workshop, 67% of residents initially reported feeling "very confident" or "fairly confident" in their patient handoff skills. After the completion of the workshops, 98% of residents reported "fairly confident" or "very confident" in their ability to perform handoff. Pre- and post-workshop surveys demonstrated self-perceived increases in confidence (P<0.001). Following the completion of the workshops, we conducted observations and found that residents properly executed handoffs, and we received no further patient safety alerts regarding communication breakdowns.

Conclusions: We identified several protocol failures in effective handoff among pediatric residents. Chief resident-led targeted workshops addressed these lapses, improved the effectiveness of patient handoffs, and reduced patient safety events related to breakdowns in communication. Our interventions increased confidence in handoff among pediatric residents, and these effects were sustained over time.

## Introduction

The Accreditation Council for Graduate Medical Education (ACGME) Pediatric Residency Program Requirements mandate that residency programs ensure effective handoff or transfer of patient care processes to facilitate continuity of care and patient safety [1]. Ineffective handoff during patient care transitions is a major cause of serious medical errors, as inaccurate, incomplete, and omitted data create uncertainties between providers. The Joint Commission reports that nearly 67% of communication errors are related to ineffective handoff of patient care [2]. Numerous studies have highlighted the harmful consequence of inadequate handoff procedures on patient safety and the increased potential for sentinel events [3-6]. 

Resident physicians conduct handoffs in all patient settings, and the frequency of patient transitions has increased since the implementation of the 80-hour workweek [7]. Standardized tools and handoff bundles have been developed to minimize medical errors during these transitions. One such bundle is the I-PASS verbal handoff program. The I-PASS mnemonic represents a standardized method of communication, with "I-PASS" standing for illness severity, patient summary, action list, situation awareness and contingency planning, and synthesis by the receiver. Implementation of the I-PASS mnemonic has been shown to improve patient safety and communication in pediatrics and has become the preferred handoff tool for many organizations [8,9].

While standardized handoff procedures such as I-PASS have proven to be successful in reducing adverse events, the resident physicians' skill, experience, and knowledge regarding the use of the tool and consistent implementation of all components play a key role in its success. Educating residents in effective handoff procedures has multiple benefits, including promoting patient safety, preventing adverse outcomes, and maintaining continuity of care [5,8-11]. Improving residents' handoff skills also fosters teamwork and enables graduate medical education programs to comply with ACGME regulations. As chief residents and residency program leadership, we were notified of an increase in patient safety alerts and delays in patient care due to presumed communication failures and gaps during handoff.

The purpose of this study was to identify areas for improvement and implement strategies to improve competence in handoff among pediatric residents. We also explored pediatric residents' confidence levels regarding handoff procedures and the effectiveness of our interventions in the transfer of care.

## Materials and methods

This study was conducted at the Valley Children's Healthcare Pediatric Residency program in Madera, California, from September 2023 to March 2024. This study was deemed exempt by the Institutional Review Board of Valley Children's Healthcare (IRB Determination-HSC2676).

Our institution utilizes I-PASS for resident handoff. All residents are trained in the adaptation of the I-PASS mnemonic, handoff processes, written handoff documents, and performance evaluation methods. Training is performed during intern orientation and annually.

We developed a 10-item handoff checklist that included components for an effective handoff. The handoff checklist included the post-graduate year (PGY) training of residents, time of observation, whether the I-PASS format was followed, and if the handoff began with the sickest patients. We also included an area to document errors in providing and receiving handoff, interruptions, and distractions, the accuracy of action items, and the provision of contingencies. We (Y.M. and M.M.) performed direct handoff observation of the resident teams during inpatient service transfer of care over six months. Residents were scored using the handoff checklist, and formative feedback was provided after each observation session. Deficits and barriers to properly executed transfer of care were noted.

Two chief residents developed a series of small group handoff workshops. Two chief residents scheduled meetings with residents of varying levels of experience to review their workflow and discuss perceived challenges together. The meetings were scheduled during protected academic time to prevent conflicts with clinical duties. The chief resident then provided participants with several cases with increasing complexity, including transfers to and from the pediatric intensive care unit and admissions from the emergency room. Subsequently, they reviewed key structured communication techniques and the oral and written components of the I-PASS mnemonic. Each team was asked to reflect on their handoff, share perceived barriers, and develop strategies to overcome these barriers. Program leadership closely monitored for patient safety alerts regarding resident handoff.

Pre- and post-workshop confidence in handoff skills was calculated from an average of each five-point Likert scale item (1=not at all confident, 5=very confident). Descriptive analyses, including counts and percentages, were completed for nominal variables, whereas mean values and standard deviations were performed for continuous variables. Data from the respondents were analyzed using chi-squared tests to assess the analytical significance of the pre- and post-activity.

## Results

We assessed 40 pediatric residents performing handoff while working on the inpatient service teams. This included 14 PGY-1, 13 PGY-2, and 13 PGY-3 residents. We observed 38 handoff sessions. All sessions were face-to-face interactions with both verbal and written communication in the I-PASS format and allowed for the receiver of the information to clarify issues and ask questions.

Protocol failures were identified in 50% of the observed handoff sessions. This included disruptions during handoff (5%) that primarily included pages and telephone calls. We also recorded incorrect relay of patient information (26%), failure to prioritize sick patients (26%), and omission of care tasks or action items (10%). The most common lapse in the handoff process was the lack of contingency planning (31%). More than one type of protocol failure was observed in 10% of handoff sessions. See Table 1. 

**Table 1:** Protocol failure observed by chief residents during handoffs of patients More than one protocol failure was observed in some categories

Protocol failures observed	n=38 (%)
Disruptions during handoff	2 (5)
Incorrect relay of information	10 (26)
Failure to prioritize sick patients	10 (26)
Omission of care tasks	4 (11)
Provision of contingency planning	12 (32)

We asked residents about their confidence level with handoff procedures. Sixty-seven percent of residents stated that they felt "very confident" or "fairly confident" that their sign-out contained adequate information for the receiving team to take care of their patients. However, 33% of residents cited feeling "not at all" or "slightly confident" with the statement "I am comfortable with the accuracy of the information received during the handoff from my peers." Additionally, 17% reported discomfort with assuming care for patients signed out to them by their peers. Regarding their comfort level in communicating contingency plans, 17% of residents reported being "not at all confident" when providing contingency plans.

Forty residents participated in the handoff workshops. The two chief residents facilitated 30-minute interactive exercises for four residents per session, and all 40 residents were able to practice the I-PASS handoff technique under chief supervision. They ensured that the sessions focused on the particular lapses identified during the initial observations.

**Figure 1:**
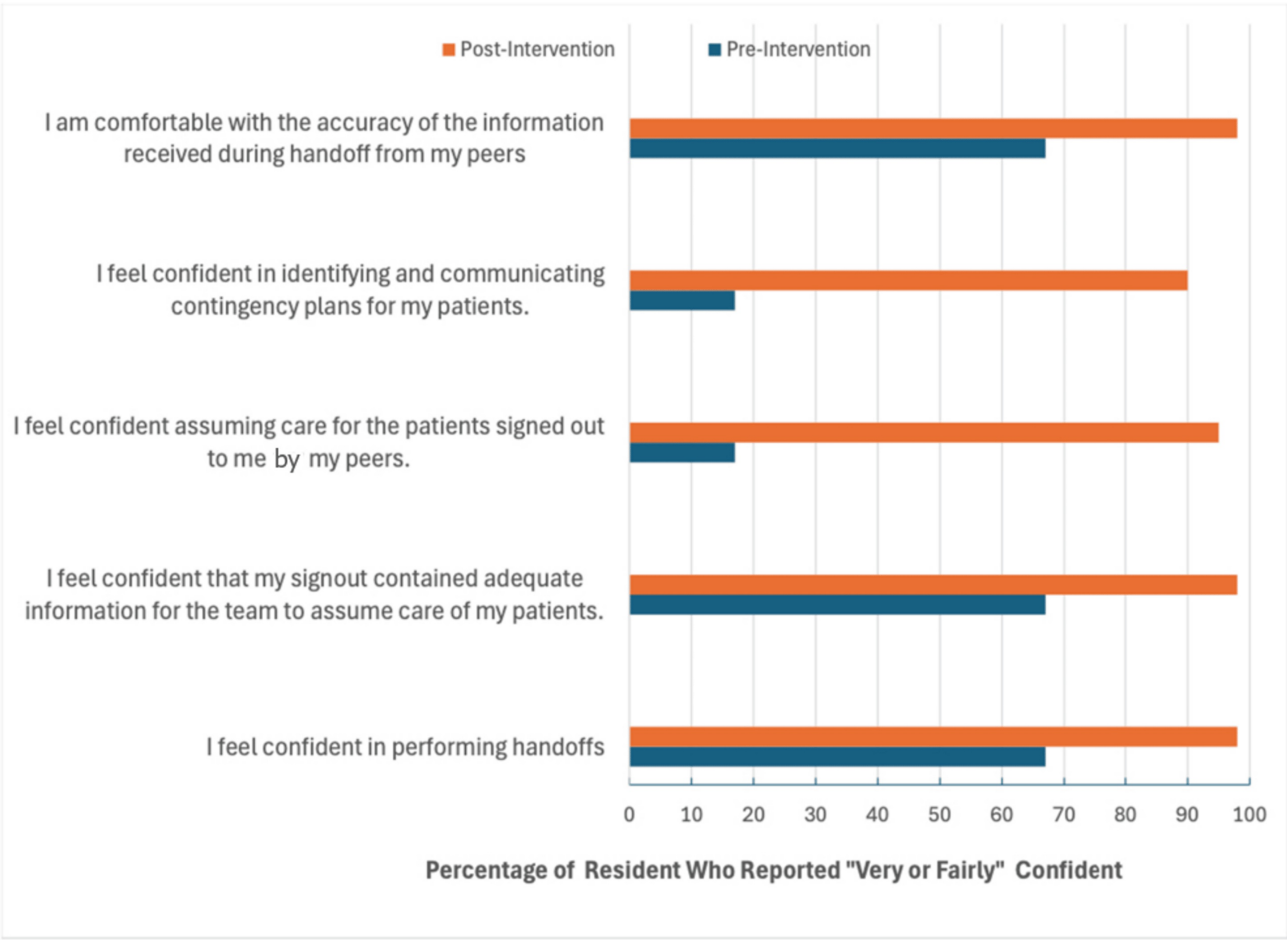
Reported level of confidence among residents regarding transfer of care pre- and post-intervention

Participants expressed a high degree of satisfaction with the small-group workshops. Eighty-five percent of participants felt that chief resident supervision of the handoff process was "very useful" or "extremely useful." Senior residents reported that they felt more empowered to give junior residents targeted feedback after the sessions. Regarding confidence in handoff before the workshop, 67% of residents felt either "not at all confident" or "not very confident." Following the completion of the workshops, 98% of residents reported feeling "fairly confident" or "very confident" in handoff (P<0.001). Pre- and post-workshop surveys demonstrated self-perceived increases in confidence in handoff procedures. Results are summarized in Figure 1. 

After the completion of our interventions, based on the availability of the chief residents, we performed random audits of resident handoffs and found no protocol failures. Additionally, we did not receive any further patient safety alerts regarding communication breakdowns at six months following the completion of this intervention.

## Discussion

The interventions in our study were successful in improving handoff practices among pediatric residents at our institution and resulted in a decline in patient safety alerts related to resident handoff. We identified several critical lapses in the effective handoff among pediatric residents. The most common protocol failures in I-PASS procedures included the provision of contingency planning, the relay of incorrect information, and a failure to prioritize sick patients. Multiple studies have shown that although residents recognize that the I-PASS program improves patient safety, they still have low levels of adherence to all five elements of the I-PASS mnemonic [12-14].

The factors contributing to incomplete or ineffective handoff are multifactorial [12-14]. In a study by Helms et al., 40% of residents did not expect to make many decisions about patients when cross-covering, and the authors suggested that residents may have a lack of ownership due to shift work mentality during these shifts [15]. This mindset may contribute to the failure to provide a clear contingency plan to handoff recipients and warrants further investigation. Similarly, Brannen et al. found that residents had varying levels of agreement about the severity of their patient's illnesses [16]. The authors highlighted the need to develop a shared understanding between physicians about the most severe problems. Emphasis on the quality of written handoff can improve the accuracy of verbal handoff and may especially be beneficial for interns as they may not be as experienced as their senior residents in identifying sick patients [16,17]. In our study, we were able to demonstrate that by targeting efforts toward the specific lapses identified during observations and providing point-of-care education, many of the protocol failures can be mitigated.

We found that direct observation by the chief residents coupled with an intervention that provided constructive and actionable feedback was successful in increasing adherence to the I-PASS bundle and confidence in the residents' ability to perform effective handoffs. Although traditionally, chief residents have primarily served as educators, their role has been expanding to include additional institutional responsibilities and initiatives. Given their unique positions as liaisons between residents and the program, chief residents may serve as the champions in shaping the transfer of care and safety initiatives through supervision, training, and curriculum delivery and oversight. Temsah et al. describe the use of video conferencing as a platform for handover in the pediatric intensive care unit [18]. Incorporating video conferencing or remote handover monitoring by chief residents may provide another area for future research. Cox et al. have also reported the value of chief resident-led initiatives in institutional quality improvement efforts [19]. As chief residents have a considerable rapport with residents, there is a potential that being observed by chiefs rather than faculty may mitigate the Hawthorne effect that has been reported in previous handoff research [14-16]. Additionally, given their clinical duties and significant resident oversight, chief residents are also better positioned to easily identify knowledge gaps in handoff skills and gather input from residents regarding their ideas for improvement [20-22].

There are several limitations to our study. It was conducted at a single, moderate-size residency program, and hence, results may not apply to larger programs. For larger residency programs, it may be challenging for chief residents alone to monitor all residents over a timely period. However, we envision that once the framework has been established, senior residents will also be able to perform observations and provide feedback. Our study evaluated only the transitions that occurred in the inpatient setting, and more work is needed in the outpatient and critical care areas. Larger studies are needed at smaller and larger residency programs as the barriers to effective handoff may differ.

## Conclusions

This study identified several critical lapses in the effective handoff among residents at our institution. Chief resident-led handoff workshops addressed these protocol failures, improved the effectiveness of patient handoffs, and were highly effective in increasing confidence in handoffs among our residents. The interventions also reduced patient safety events related to breakdowns in communication, and the effect was sustained. Residents' feedback on the experience and their suggestions for modifications of the workshops could be incorporated into future refinements of this program.

Our findings also emphasize the crucial role that chief residents play in identifying and addressing the educational priorities of resident physicians and optimizing patient safety. Additionally, we provide useful insights for medical education leaders planning to implement a handoff improvement program. Further studies are needed to identify different observation tools for chief residents to consider when developing handoff workshops. The use of artificial intelligence-generated written handoffs and the use of recorded video handoffs for trainee educational interventions may be areas for potential study.
